# Seeking for Innovation with Magnetic Resonance Imaging Paramagnetic Contrast Agents: Relaxation Enhancement via Weak and Dynamic Electrostatic Interactions with Positively Charged Groups on Endogenous Macromolecules

**DOI:** 10.1021/jacs.3c06275

**Published:** 2023-12-28

**Authors:** Rachele Stefania, Lorenzo Palagi, Enza Di Gregorio, Giuseppe Ferrauto, Valentina Dinatale, Silvio Aime, Eliana Gianolio

**Affiliations:** † Department of Molecular Biotechnology and Health Sciences, University of Torino, Torino 10126, Italy; ‡ Department of Science and Technological Innovation, University of Eastern Piedmont, Alessandria 15120, Italy; § IRCCS SDN SYNLAB, Napoli 80142, Italy

## Abstract

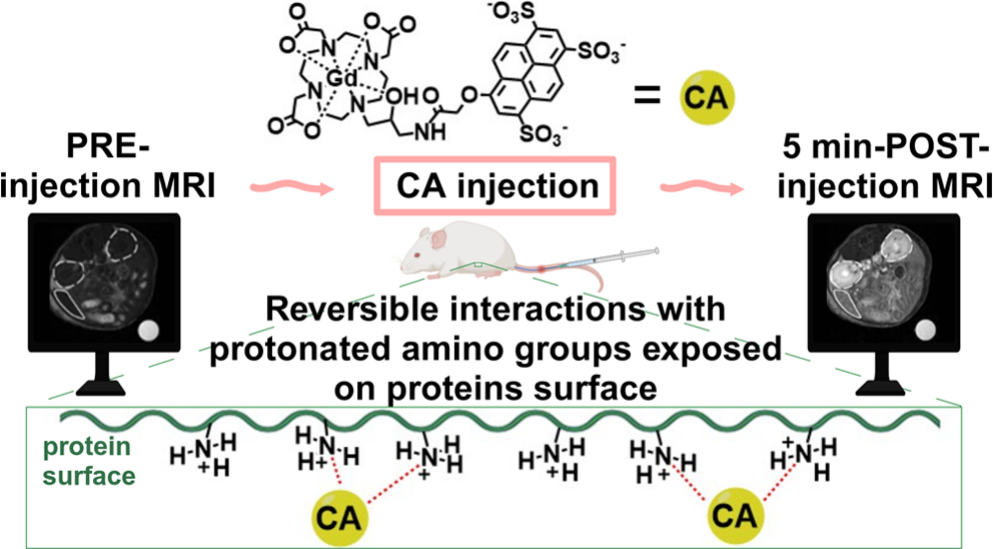

Gd-L1 is a macrocyclic Gd-HPDO3A derivative functionalized with a short spacer to a trisulfonated pyrene. When compared to Gd-HPDO3A, the increased relaxivity appears to be determined by both the higher molecular weight and the occurrence of an intramolecularly catalyzed prototropic exchange of the coordinated OH moiety. In water, Gd-L1 displayed a relaxivity of 7.1 mM^–1^ s^–1^ (at 298 K and 0.5 T), slightly increasing with the concentration likely due to the onset of intermolecular aggregation. A remarkably high and concentration-dependent relaxivity was measured in human serum (up to 26.5 mM^–1^ s^–1^ at the lowest tested concentration of 0.005 mM). The acquisition of ^1^H-nuclear magnetic relaxation dispersion (NMRD) and ^17^O-*R*_2_ vs *T* profiles allowed to get an in-depth characterization of the system. *In vitro* experiments in the presence of human serum albumin, γ-globulins, and polylysine, as well as using media mimicking the extracellular matrix, provided strong support to the view that the trisulfonated pyrene fosters binding interactions with the exposed positive groups on the surface of proteins, responsible for a remarkable *in vivo* hyperintensity in *T*_1w_ MR images. The *in vivo* MR images of the liver, kidneys, and spleen showed a marked contrast enhancement in the first 10 min after the i.v. injection of Gd-L1, which was 2–6-fold higher than that for Gd-HPDO3A, while maintaining a very similar excretion behavior. These findings may pave the way to an improved design of MRI GBCAs, for the first time, based on the setup of weak and dynamic interactions with abundant positive groups on serum and ECM proteins.

## Introduction

Contrast agents (CAs) play a crucial role in diagnostic magnetic resonance imaging (MRI) as they add relevant physiological information to the outstanding anatomical resolution of the images acquired with this modality.^1−7^ They usually consist of paramagnetic gadolinium complexes that cause a marked reduction in the proton relaxation times in the regions where they distribute. Their efficiency is first of all represented by their relaxivity (*r*_1_), i.e., the relaxation enhancement brought to solvent water protons at a concentration of 1 mM.^8,9^ Over the years, probe developers have paid great attention to improving the relaxivity of gadolinium-based contrast agents (GBCAs),^10,11^ in particular, by optimizing their structures in terms of the number and exchange rate of the coordinated water molecules in the inner and in the second coordination sphere^12−14^ and by controlling the molecular reorientational time through the introduction of substituents on the surface of the ligand capable of either increasing their molecular weight (MW)^15^ or setting up suitable interactions with macromolecules.^10,16−18^ Often, the target macromolecule is represented by human serum albumin (HSA)^19,20^ thus endowing the GBCA with blood pool properties, with a primary aim of application in the field of the acquisition of angiographic images.^21−24^ In most cases, the binding to HSA has been pursued through the introduction of substituents capable of recognizinng the well-known binding sites for lipophilic drugs usually identified as the Sudlow sites.^25,26^

Our aim is to seek new routes for the generation of relaxation enhancement through the involvement of novel types of noncovalent interactions with endogenous systems. In this work, we report on our recent observations involving the reversible setup of binding motifs with abundant positively charged groups exposed on the outer surface of endogenous proteins. In principle, the protonated functionalities provide the source for the setup of electrostatic salt bridges and cation−π interactions. They are known to occur in several biological systems to yield important contributions for instance to generate robust wet adhesion and cohesion in humid/underwater environments.^27^ Cation−π interactions are essentially of electrostatic origin because a positively charged cation interacts with the negatively charged electron cloud of π systems and their strengths stand out as stronger than typical hydrogen bonds.^28^ One may surmise that HSA (which contains a high number of positively charged NH_3_^+^ lysine residues)^29^ and γ-globulins, endowed with a high isoelectric point,^30^ could be considered potential substrates for the setup of binding interactions based on the presence of protonated amino groups.^31−33^

In this context, we synthesized a GBCA bearing on its surface the trisulfonated pyrene derivative of 8-hydroxypyrene-1,3,6-trisulfonic acid (HPTS, pyranine), and we undertook the study of its interaction with macromolecules exposing cationic amino groups on their surface. The presence of a large π system is expected to be beneficial for the setup of cation-π interactions, whereas the three negatively charged sulfonated groups could be involved in the formation of salt bridges with protonated amino groups.

## Results

### Synthesis of Gd-L1 and Eu-L1

**Scheme 1:**
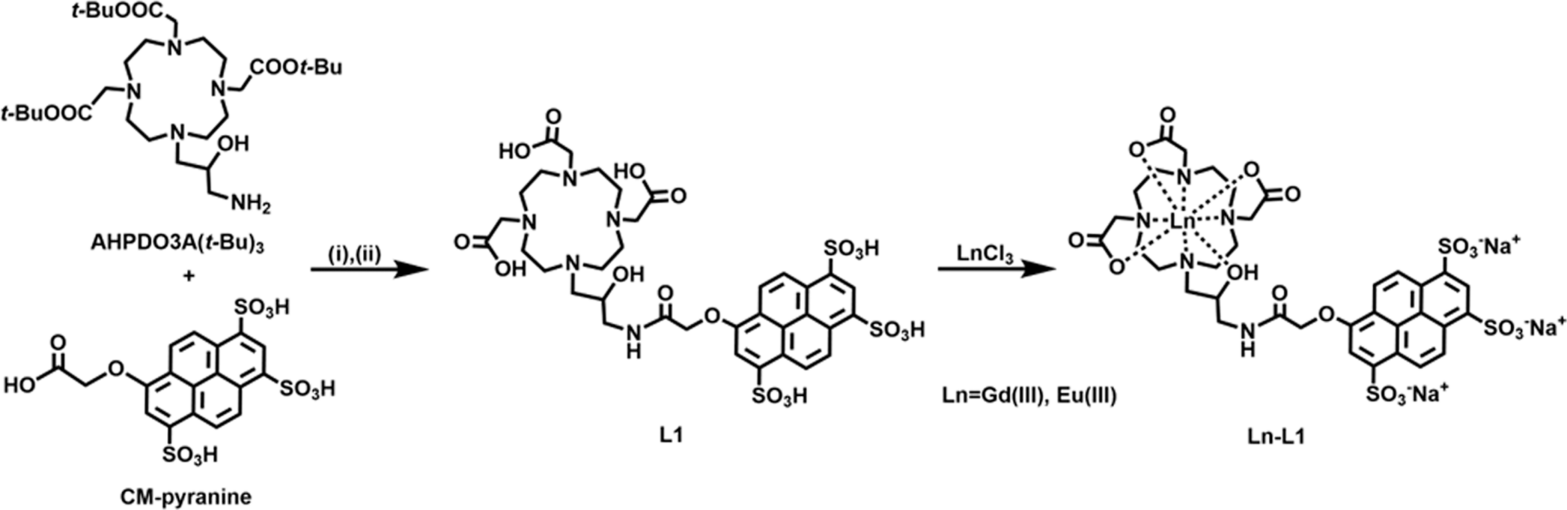
Synthesis of the L1 Ligand and Its Ln(III) (Gd(III) or Eu(III)) Complexes Reagents and conditions: (i) HBTU, DMF, DIPEA (ii) TFA, DCM 1/4 v/v.

The synthesis of Ln-L1 is shown in Scheme 1 (Ln = Gd or Eu). Ligand L1 was obtained by coupling 8-O-carboxymethylpyranine (CM-pyranine) to amino-functionalized AHPDO3A(*t*-Bu)_3_ using HBTU (*o*-(benzotriazol-1-yl)-*N*,*N*,*N*′,*N*′-tetramethyluroniumhexafluorophosphate) and DIPEA (*N,N*-diisopropylethylamine) in DMF, followed by the deprotection of *t*-butyl esters in the presence of TFA (trifluoroacetic acid) with an overall yield of about 50%. The protected AHPDO3A(*t*-Bu)_3_ ligand was synthesized as reported in the literature;^34^ briefly, *N*-Cbz-2,3-epoxypropylamine was opened by the secondary amine of DO3A-(O-*t*-Bu)_3_ and then the Cbz group was removed by Pd/C catalyzed hydrogenolysis. Conversely, CM-pyranine was prepared by the alkylation of the commercially available HPTS with methyl bromoacetate in refluxing methanol. Then, the obtained methyl ester was quantitatively hydrolyzed with 2.4 M aqueous HCl at 90 °C.^35^ The final L1 ligand was purified by chromatography on an AmberChrom resin with 46% yield. The corresponding Ln(III) complexes (Ln(III) = Gd(III) and Eu(III)) were then prepared by mixing stoichiometric amounts of L1 and LnCl_3_ at pH 6.7 in water. Upon removal of the formed salts, Ln-L1 complexes were obtained at an excellent purity level. The ^1^H NMR spectrum (Figure S7) of Eu-L1 showed the presence of the two expected diastereoisomers, namely TSAP (twisted square anti-prismatic) and SAP (square anti-prismatic) in the ratio of 3:2, respectively.^36−38^

### *In Vitro* Relaxometric Studies

**Figure 1:**
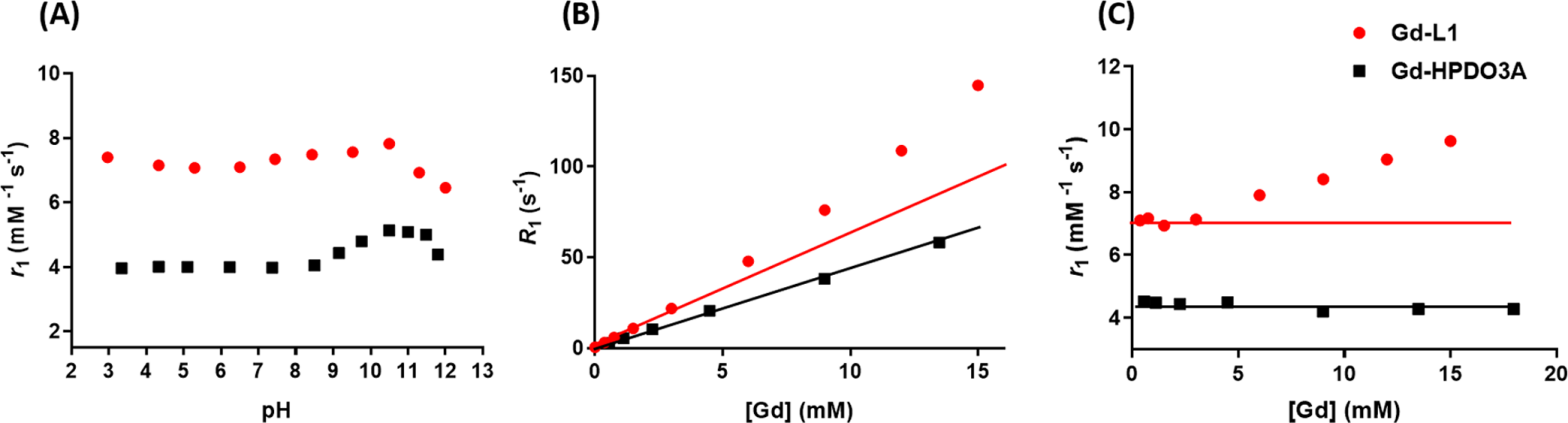
(A) Millimolar relaxivity values (*r*_1_) of Gd-L1 and Gd-HPDO3A in bidistilled water as a function of solution pH. (B) Relaxation rates (*R*_1_) in bidistilled water as a function of the Gd-L1 or Gd-HPDO3A concentration at neutral pH. (C) Millimolar relaxivity values (*r*_1_) calculated from the observed relaxation rates reported in (B) and those normalized to the Gd-L1 concentration in the same concentration range and neutral pH. All data were measured at 298 K and 21.5 MHz for Gd-L1 (red circles) and Gd-HPDO3A (black squares).

In neat water, the measurement of the relaxation rates, *R*_1_, of a 1 mM solution of Gd-L1 as a function of pH (at 21.5 MHz and 298 K) yielded a relaxivity value of 7.1 mM^–1^ s^–1^ which remained almost constant upon increasing the pH to 9–10 showing a slight decrease at higher pH values (Figure 1A).

This finding indicates a substantial difference with respect to the pH dependence of the relaxivity of the parent Gd-HPDO3A, which shows a more pronounced increase at basic pH due to the mobilization of the coordinated hydroxyl proton.^39^ At neutral pH, upon an increase in the Gd-L1 concentration (Figure 1B), the relaxation rate did not show a linear increase. This deviation from the linearity is also clearly reflected in the relaxivity plot (Figure 1C), thus revealing that the relaxivity is not constant by varying the Gd-L1 complex concentration. The observed behavior suggests the occurrence of a weak intermolecular self-aggregation among Gd-L1 molecules likely as a consequence of the interaction established between the pyrene-containing functionality and the tetra-aza macrocycle of Gd-HPDO3A, as recently reported.^40^ This behavior is consistent with the assumption that the linking arm is flexible enough to allow the setup of this interaction. Conversely, the parent Gd-HPDO3A, as expected, showed a linear increase in *R*_1_ as the concentration of the paramagnetic complex increased (Figure 1B) with *r*_1_ remaining constant throughout the entire range of examined concentrations (Figure 1C).

**Figure 2:**
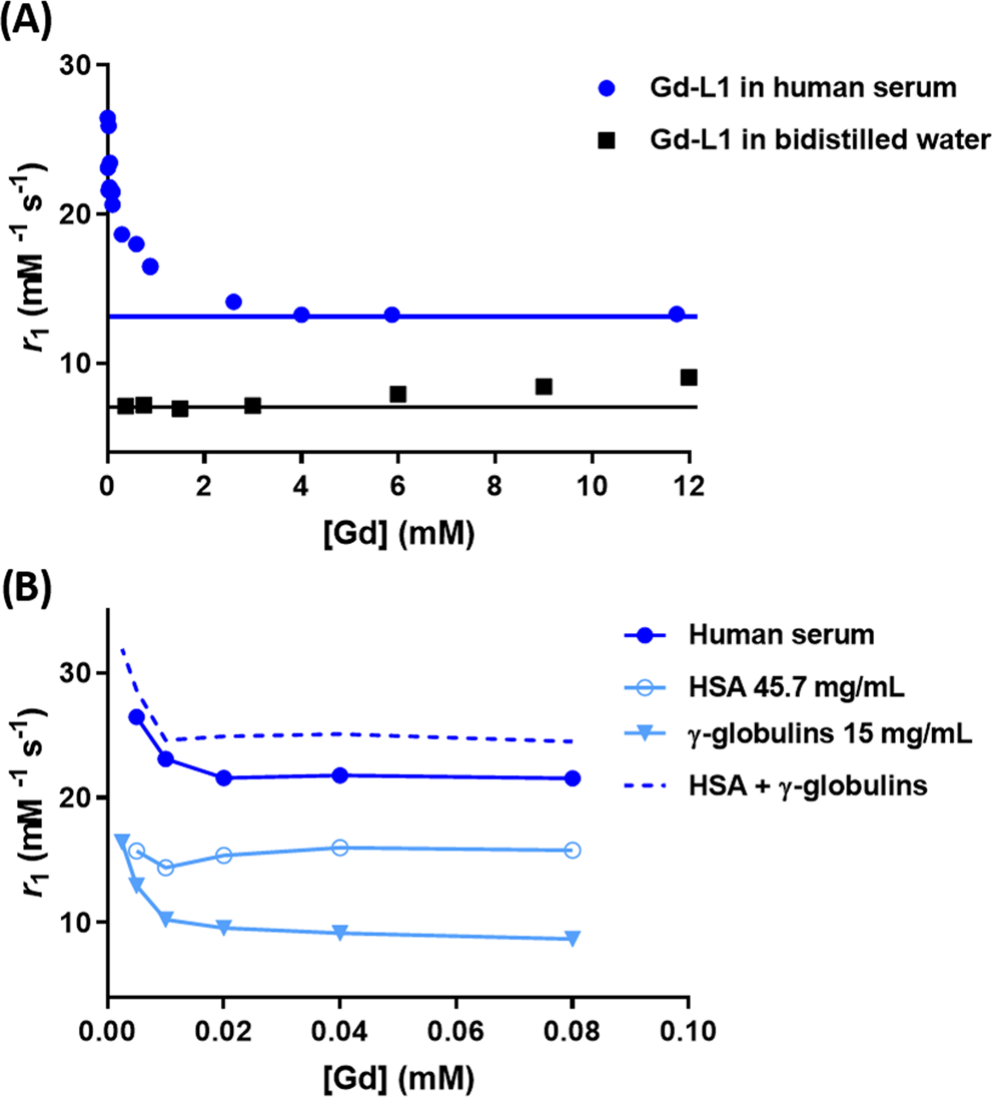
(A) Millimolar relaxivity values of Gd-L1 in human serum and in pure bidistilled water at neutral pH in the concentration range of 0.005–12 mM. (B) Expansion of the low concentration range data (0.005–0.08 mM) reported in (A) with the addition of relaxivity data obtained in PBS solutions containing HSA (45.7 mg/mL) and γ-globulins (15 mg/mL). The dotted line represents the calculated profile obtained by summing the relaxivities measured in the media containing γ-globulins and HSA, respectively. Data were acquired at 298 K and 21.5 MHz.

In human serum the relaxivity of Gd-L1 displayed markedly higher values than in pure water, and it remained almost constant from 2 to 12 mM (Figure 2A).

Interestingly, and differently from what is observed in water, the observed relaxivity values of Gd-L1 in serum markedly increased upon decreasing the concentration of Gd-L1 below 1 mM to reach very high values in the micromolar range of concentrations (Figure 2A). The relaxivity of the parent Gd-HPDO3A in human serum was found to be equal to 5.7 ± 0.36 mM^–1^ s^–1^, a value that remained constant across the entire investigated concentration range. This value is slightly higher than the relaxivity measured in water (Figure 1C). The difference may be attributed to the higher viscosity of the serum and/or to the occurrence of a contribution from the prototropic exchange of the coordinated OH moiety. To get more insight into the observed behavior of Gd-L1 in serum, it was deemed of interest to focus our attention on the measure of the relaxivity at concentrations lower than 0.1 mM (Figure 2B). To this end, the separate contributions from serum albumin and γ-globulins (both used at their standard concentration in serum),^41^ which together make up three-quarters of the total serum proteins, were investigated in the low Gd-L1 concentration range. In PBS, in the presence of a physiological HSA concentration, the relaxivity was almost constant at ca. 15.8 mM^–1^ s^–1^ over the explored range of concentrations (5–80 μM). The proton relaxation enhancement (PRE) titration^42^ yielded the following binding parameters: n*K*_a_ = 489 M^–1^ and *r*_b_ = 39.8 mM^–1^ s^–1^ (Figure S4A). The obtained association constant allows one to calculate that, under the experimental conditions applied for the experiment reported in Figure 2B (i.e., [HSA] = 0.6 mM, [Gd-L1] = 5–80 μM), around 22% of the Gd complex is bound to HSA. Next, the relaxivity was measured for γ-globulins containing solutions (15 mg/mL) in the same Gd-L1 concentration range as applied for HSA. In this case, the relaxivity enhancement attained the values shown with HSA only at very low Gd-L1 concentration. The concentration of γ-globulins in serum is much lower (ca. 0.1 mM) than that of albumin (0.6 mM). A PRE titration carried out at the Gd-L1 concentration of 50 μM and variable concentration of γ-globulins yielded the following results: n*K*_a_ = 56 M^–1^, *r*_b_ = 47.8 mM^–1^ s^–1^ (Figure S4B). In the considered Gd-L1 concentration range, the sum of the relaxivities measured for HSA and γ-globulin-containing solutions, at their physiologic concentrations, yielded a profile (dotted line in Figure 2B) that nicely parallels the one observed in the case of whole blood serum.

In order to get more insight into the possible occurrence of a hydrophobic interaction in the binding scheme of Gd-L1 on HSA, the relaxation rates of solutions containing the paramagnetic complex (0.1 mM) and the serum protein (0.6 mM) were measured in the presence of the well-established binders (0.6 mM) for the Sudlow sites I (subdomain IIA) and II (subdomain IIIA) and for subdomain IB.^25,26,43,44^ The results obtained from the competition test (Figure S5) clearly show that the relaxation enhancement brought by Gd-L1 upon binding to HSA is almost unaffected by the presence of the strong binders at the typical hydrophobic sites of HSA, thus ruling out the possibility that hydrophobic interactions play a role in the binding of Gd-L1 to HSA. These findings support the view that the presence of three sulfonated groups on the external perimeter of the pyrene moiety induces a marked hydrophilicity hampering its binding to the hydrophobic binding sites on HSA. Support for the surmised good hydrophilicity of Gd-L1 has been gained by measuring its log *P*, which was found to be quite low (log *P* = −2.28). Conversely, the competition test with HPTS showed a decrease in relaxivity up to a value close to that of the free form, as expected in the case of the two species competing for the same sites on the protein. Actually, the binding affinity of HPTS was higher than the one shown by Gd-L1 being *K*_a_ = 1.8 × 10^4^ M^–1^ (Figure S6).

**Figure 3:**
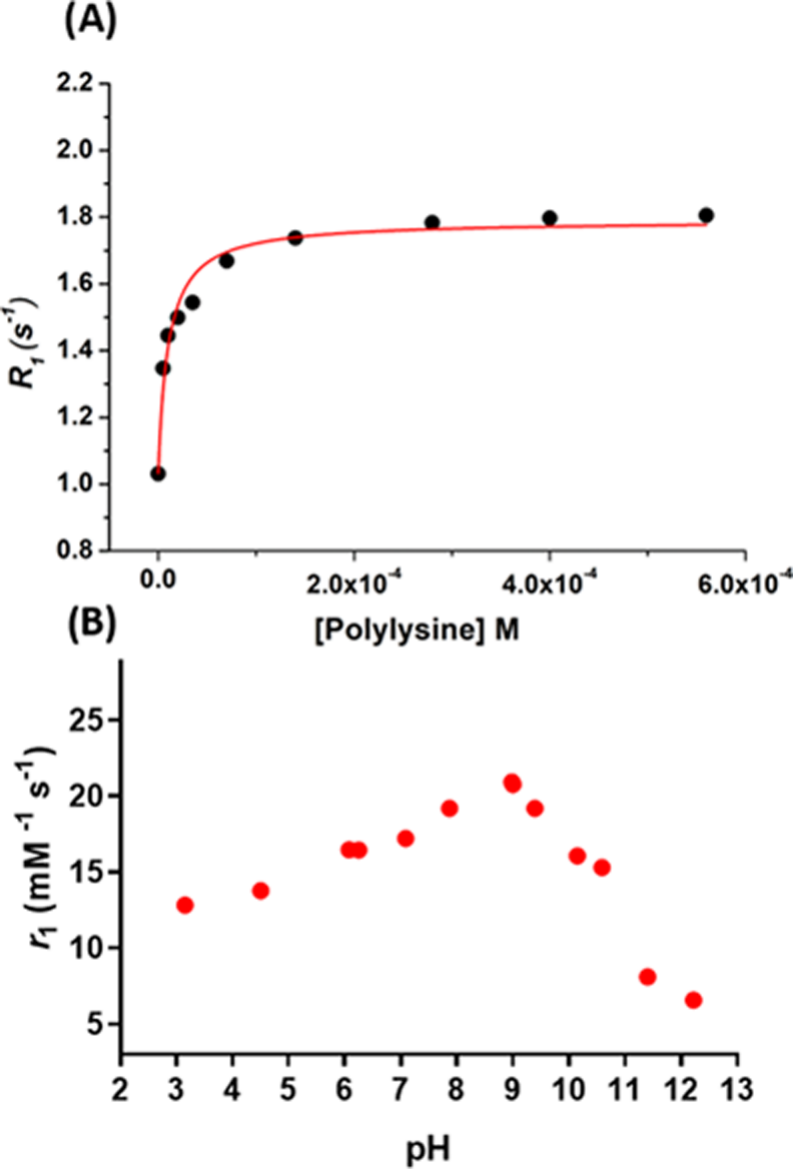
(A) Relaxation rate of a 0.1 mM solution of Gd-L1 in the presence of increasing amounts of polylysine (average MW: 50 kDa). Data measured at 298 K and 21.5 MHz, in PBS. (B) Millimolar relaxivity values of Gd-L1 in the presence of polylysine as a function of the solution pH. The relaxation rates were measured with 0.1 mM Gd-L1 and 0.3 mM polylysine and normalized to 1 mM Gd concentration.

The similarities observed in the behavior of Gd-L1 relaxivity in the presence of HSA and γ-globulins led us to surmise that the observed relaxation enhancement may involve the interaction with NH_3_^+^ moieties exposed on the protein surfaces. To get more insight into this possibility we went to consider polylysine as a good model for investigating the role of amino groups on the surface of macromolecules. In the presence of 0.1 mM polylysine (MW = 30–70 kDa) the relaxivity of Gd-L1 increased up to ca. 13 mM^–1^ s^–1^, in PBS at 298 K and 21.5 MHz. The titration of Gd-L1 (0.1 mM) with increasing amounts of polylysine (concentration estimated on an average MW of 50 kDa) yielded an n*K*_a_ value of 1 × 10^5^ M^–1^ and a relaxivity of the supramolecular adduct (*r*_b_) of 15.3 mM^–1^ s^–1^ (Figure 3A). *r*_1_ was dependent on the pH of the solution showing a steady increase to reach the maximum value of ca. 21 mM^–1^ s^–1^ at pH 9 (Figure 3B).

**Figure 4:**
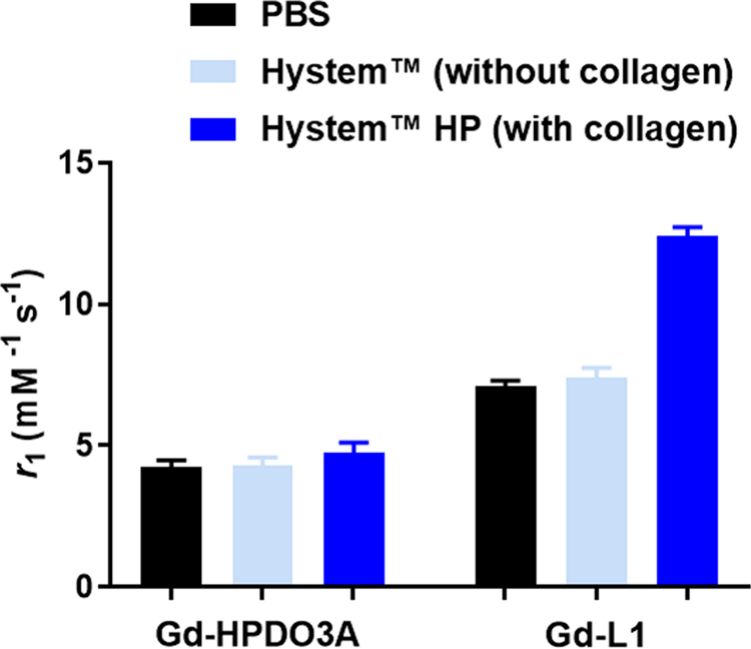
Comparison of the millimolar relaxivity values of Gd-HPDO3A and Gd-L1 in PBS and in Hystem with and without the collagen component. The relaxation rates were measured with the 0.1 mM Gd complex and normalized to 1 mM. Data were acquired at 298 K and 21.5 MHz.

On the basis of these results, the next step dealt with the study of the relaxometric properties of Gd-L1 in a medium mimicking the extracellular matrix (ECM) whose collagen proteins are rich in NH_3_^+^ exposed residues.^45^ A material considered a good model of the ECM is Hystem, a complex hyaluronic acid-based hydrogel matrix, which closely mimics the complex tridimensional extracellular environment found in living systems.^46^ Hystem is commercially available in formulations with or without collagen. In the collagen-containing medium, at 21.5 MHz and 298 K, Gd-L1 yielded a relaxivity of 12.4 mM^–1^ s^–1^ which decreased to ca. 7 in the collagen-deprived product (Figure 4). As a control, the relaxivity of Gd-HPDO3A, measured under the same conditions, displayed only minor differences with respect to PBS, either in the presence or in the absence of collagen.

The different behaviors of the two paramagnetic complexes clearly suggest that Gd-L1 is interacting with collagen. These fibrotic proteins are rich in lysine residues, and they are therefore good candidates for acting as target sites for Gd-L1 in the ECM.

**Figure 5:**
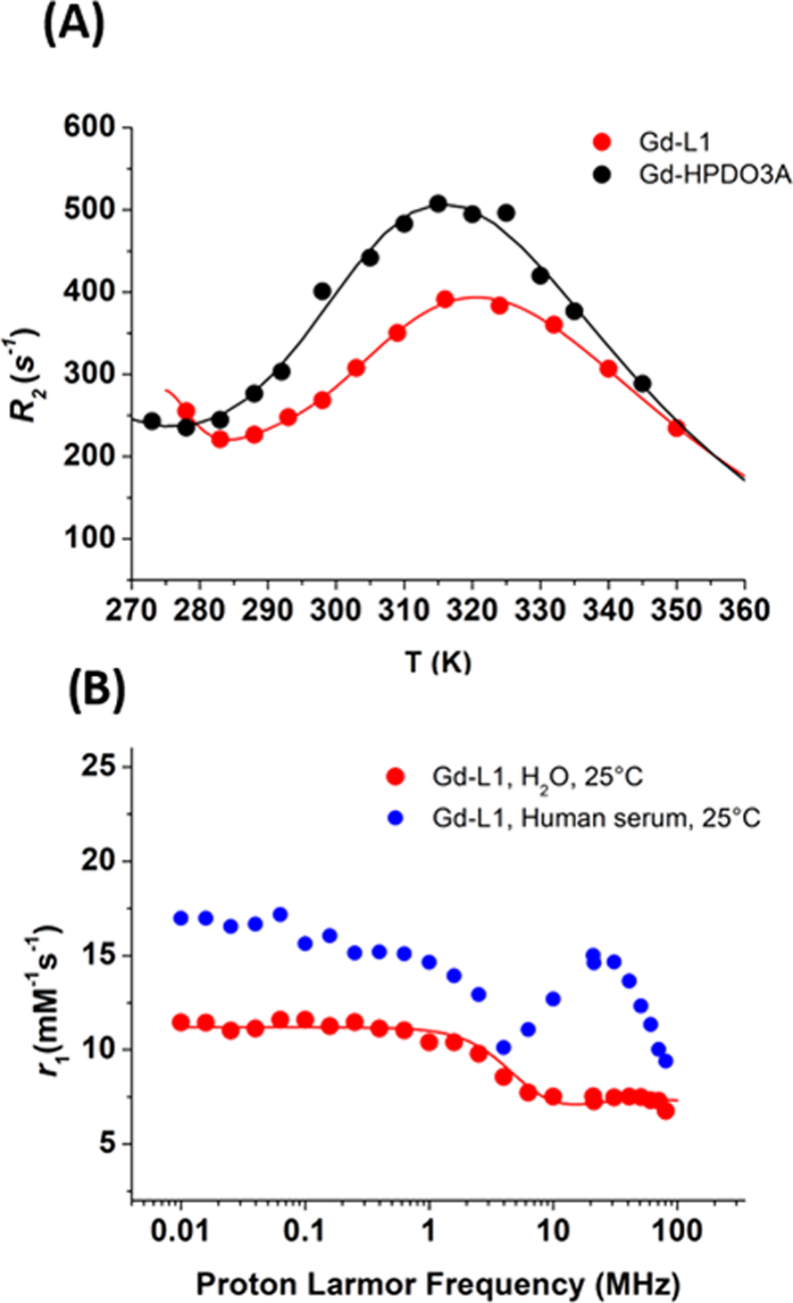
(A) ^17^O-*R*_2_ vs T profiles of Gd-L1 compared to that of Gd-HPDO3A measured in water on an NMR spectrometer operating at 14.1 T; data normalized to 20 mM Gd concentration. (B) ^1^H-NMRD profiles measured on solutions of Gd-L1 1 mM in water and in human serum, in the frequency range 0.01–300 MHz at 298 K. Additional relaxivity values in Hystem and Hystem-HP were also acquired over a frequency range of 20–300 MHz at 298 K in solutions of Gd-L1 0.1 mM. All data were normalized to 1 mM Gd concentration and reported as millimolar relaxivity (*r*_1_) values.

Next, the relaxometric properties of Gd-L1 were further investigated by measuring the ^17^O-*R*_2_ vs T profile in water (compared to the parent Gd-HPDO3A in Figure 5A) and the nuclear magnetic relaxation dispersion (^1^H-NMRD) profiles in water, serum, and Hystem with or without collagen (Figure 5B). The ^17^O-*R*_2_ vs T profile measured for an aqueous solution of Gd-L1 (Figure 5A) has the typical shape observed in the presence of two species/isomers in solution. As for the parent Gd-HPDO3A, they can be associated with TSAP and SAP diastereoisomers for which a TSAP/SAP ratio of 30/70 was reported.^47,48^ For Gd-L1, the isomer distribution was found to be 60% TSAP and 40% SAP, in agreement with the ratio observed for Eu-L1 (Figure S7 where the mono- and bi-dimensional ^1^H NMR spectra are reported). The analysis of the ^17^O-*R*_2_ vs T profile allowed to calculate exchange lifetimes of 1.9 and 470 ns for TSAP and SAP isomers, respectively, which resulted in a weighted average of 189 ns. Notably, the higher proportion of the TSAP isomer is responsible for a substantially shorter water exchange lifetime than the one determined for Gd-HPDO3A, where the average τ_M_ was calculated to be 450 ns.^48^ A rapid exchange of the coordinated water molecule is definitely a favorable feature for achieving high relaxivities upon binding to macromolecular systems.^20,37,38^ The ^1^H-NMRD profile of Gd-L1 in water (Figure 5B) was fitted by considering the contribution from 1.5 coordinated water molecules, i.e., one inner sphere water molecule plus the proton of the coordinating alcoholic group whose involvement in the exchange with water appears to occur already at neutral pH.

The ^1^H-NMRD profile of Gd-L1 in serum and in Hystem-HP fully supports the view of the occurrence of a binding interaction to proteins that results in prolongation of the reorientational correlation time (τ_R_) yielding a relaxivity hump at about 30 MHz. In collagen-deprived Hystem, the relaxivity is almost the same as the one measured in water.

The quantitative analysis of ^17^O-*R*_2_ vs T and ^1^H-NMRD profiles (in water) yielded the values of the relevant relaxometric parameters reported in Table 1.

**Table 1:** Relaxometric Parameters of Gd-L1 Derived from the Fitting of ^17^O-*R*_2_*vs* T and ^1^H-NMRD Data Reported in Figure 5^a^

τ_M_^SAP^ (ns)	470 ± 27.6
τ_M_^TSAP^ (ns)	1.9 ± 1.4
τ_M_^w.avg.^ (ns)	189 ± 11.2
τ_R_ (ps)	115 ± 4.6
Δ^2^ (10^19^ s^–2^)	4.89 ± 0.91
τ_v_ (ps)	19.1 ± 3.3

a For the ^117^O-*R*_2_ vs *T* fitting procedure the following parameters were fixed: *q* = 1, *r*_GdO_ = 2.5 Å, *E*_r_ = 10 kJ mol^–1^, *E*_v_ = 10 kJ mol^–1^, *A*/*h* = −3.5 × 10^6^ rad s^–1^. The ^1^H-NMRD profiles were acquired at 298 K and the following parameters were fixed during the fitting procedure: *q* = 1.5, *r*_GdH_ = 3.1 Å, *a*_GdH_ = 3.8 Å, *D*_GdH_ = 2.24 × 10–5 cm^2^ s^–1^.

The stability of Gd-L1 toward transmetalation was checked through a relaxometric competition assay with ZnCl_2_ at a temperature of 310 K.^49^ The water proton relaxation rate remained constant while the Gd-L1 solution (1 mM) was kept stirring for 7 days in the presence of 10 equiv of zinc in phosphate buffer 50 mM (Figure S8).

### *In Vivo* MRI Studies

**Figure 6:**
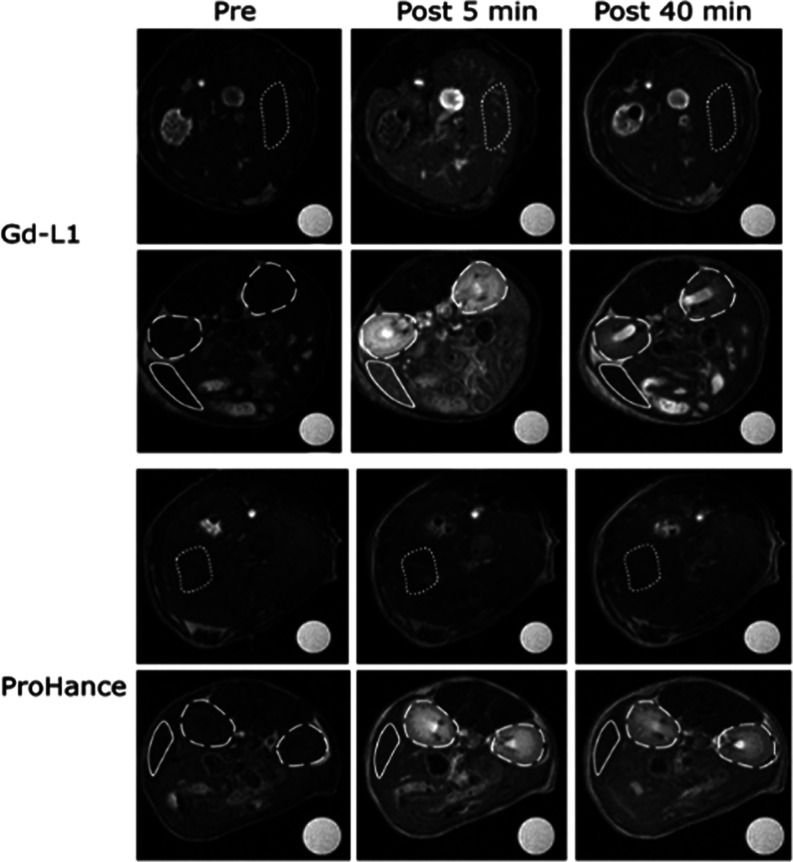
*In vivo* axial *T*_1w_-MR images (at 7.1 T) of healthy Balb/c mice *pre* and *post* (at 5 and 40 min) injection of Gd-L1 or ProHance 0.15 mmol/kg (dotted lines indicate liver ROI, dashed lines indicate kidneys ROI, and continuous lines indicate spleen ROI).

**Figure 7:**
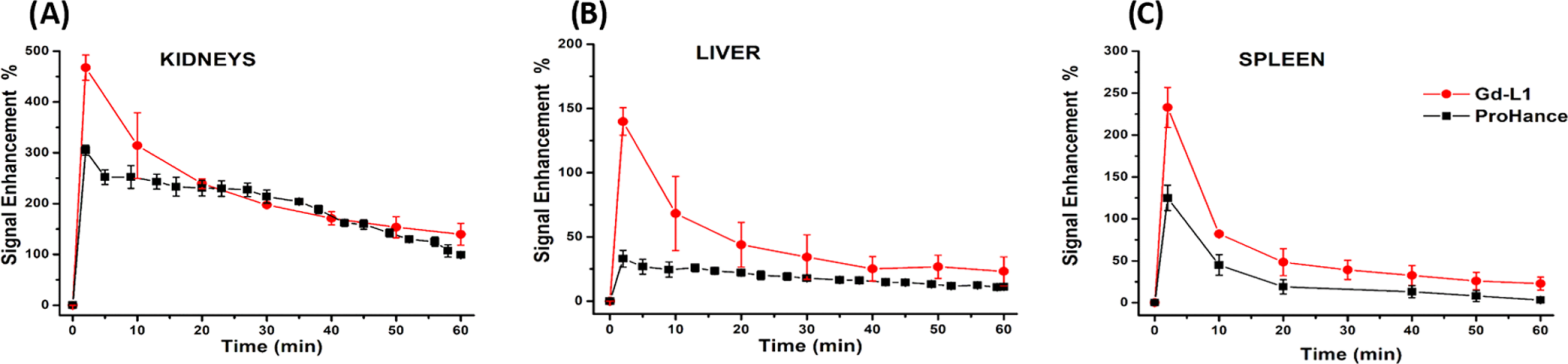
MR signal enhancement in the (A) kidneys, (B) liver, and (C) spleen of healthy Balb/c mice (*n* = 3) upon injection of Gd-L1 or ProHance 0.15 mmol/kg.

The MRI properties of Gd-L1 were investigated on healthy Balb-c mice. Very good contrast enhancements were observed in the liver, spleen, and kidneys (Figure 6). The observed maxima in contrast enhancement in *T*_1_-weigthed images were ca. 2–6-fold the values observed for ProHance (Gd-HPDO3A) administered at the same dose. Interestingly, the contrast effect induced by Gd-L1 decreased quite rapidly; in fact, 15–30 min after administration, the observed contrast enhancements were almost the same for both Gd-L1 and Gd-HPDO3A (Figure 7).

ICP-MS analyses for Gd quantification were conducted on blood samples collected from healthy mice at different time points (as shown in Figure S9) after intravenous administration of 0.15 mmol/kg contrast agent to determine the rates of elimination of Gd-L1 and ProHance from the blood. The elimination rate of Gd-L1 from the bloodstream was only slightly less rapid than that of ProHance, with elimination half-lives of 14.1 ± 1.7 and 12.1 ± 1.6 min observed for Gd-L1 and ProHance, respectively.

## Discussion

Gd-L1 was shown to be a useful model system for investigating novel kinds of interactions with endogenous proteins. It owns three residual negative charges brought at the external perimeter of the pyrene substituent. The presence of the sulfonate moieties endows the molecule with good water solubility, while the conjugated aromatic system represents an excellent source of π-electrons. Recently, we showed that the parent HPTS system can be exploited for the setup of hydrophobic interactions with the tetraazacyclododecane cage of macrocyclic Gd complexes.^40^ In the case of Gd-L1, in addition to analogous weak π-bonding intermolecular associations, the dominant binding properties appear to be driven by the interaction with positively charged residues exposed on the protein surfaces. Typically, these are represented by lysine or arginine residues in serum and collagen proteins. Gd-L1 is not expected to have any binding interaction with proteoglycans, the main components of the ECM as they are often negatively charged systems (e.g., heparan sulfate, hyaluronic acid, chondroitin sulfate, and dermatan sulfate). Possibly, there is a limited effect due to the increased viscosity, as shown by the small increase observed for the parent Gd-HPDO3A in Hystem (Figure 4).

In water, the Gd-L1 relaxivity value of 7.1 mM^–1^ s^–1^, measured for 1 mM concentration at 21.5 MHz, 298 K and neutral pH, appears in agreement with the occurrence of an intramolecular catalysis of the prototropic exchange of the coordinated alcoholic group.^50,51^ The catalysis likely involves the amide proton, as previously shown for similar systems.^52−54^ Here, the catalytic effect appears even more marked, as the contribution of the OH moiety appears evident already at neutral pH values. Of course, the increased τ_R_, due to the increased MW (with respect to the parent Gd-HPDO3A), is also important. The decrease in relaxivity observed at pH > 10 is indicative of the structural change following the deprotonation of the alcoholic moiety as previously observed for the parent Gd-HPDO3A.^39,52^ The ^17^O-*R*_2_ vs T measurement suggests the occurrence of the SAP and TSAP diastereoisomers as observed for the parent Gd-HPDO3A, with a slight increase of the TSAP form. This finding is further supported by the acquisition of the ^1^H NMR spectra of Eu-L1, which clearly showed an analogous distribution of the two diastereoisomers (Figure S7).

An interesting result dealt with the increase in *r*_1_ observed in water upon increasing the Gd-L1 concentration to suggest the reversible formation of aggregates likely based on the hydrophobic interactions between the pyrene substituent and the tetra-aza macrocycle of the Gd-HPDO3A complex. This type of interaction was previously identified in binary mixtures of Ln-HPDO3A (Ln = Gd, Eu, Yb) and HPTS.^55^ It seems reasonable to expect that the constraint introduced on the pyrene derivative containing moiety upon its binding to Gd-HPDO3A causes a weakening in the hydrophobic interaction with respect to the case of the binary mixtures of the two parent molecules. An impressive relaxivity value of 21.5 mM^–1^ s^–1^ is measured at 21.5 MHz and 298 K in human serum when the concentration of the complex is 0.1 mM. A set of *r*_1_ measurements of Gd-L1 as a function of the Gd concentration (Figure 2) clearly showed that the relaxivity in serum is markedly affected when the concentration is <0.02 mM reaching a value of 26.5 mM^–1^ s^–1^ at the lowest measured concentration (5 μM). Above 4 mM, the relaxivity in serum remained constant at a remarkable value of ca. 13.5 mM^–1^ s^–1^. The observed high relaxivity values clearly suggest that, in serum, Gd-L1 interacts with sites on the endogenous macromolecules that cause elongation of its reorientational time and, in turn, leads to the observed relaxivity enhancement. The comparison of the NMRD profiles acquired in water and in human serum (Figure 5B) unambiguously supports this view.

The marked effects on relaxivity upon decreasing the molar ratio between Gd-L1 and the interacting macromolecules (Figure 2B) can be accounted for in terms of a higher molar fraction of the bound complex form with respect to the free one (in this case, the bound forms appear also endowed with higher relaxivities). In fact, increasing the concentration of Gd-L1 over the fixed concentration of protein (0.1 mM for γ-globulins), the percentage of free complex increases, and the resulting relaxivity, that corresponds to the sum of free (low relaxivity) and bound (high relaxivity) fractions, decreases. This finding is consistent with the view that, when multiple binding sites are present, the shift favors binding sites characterized by higher binding affinity.

Upon carrying out a relaxivity vs concentration assessment with HSA in PBS, it was evident that this protein is the major pool for the setup of binding interactions of Gd-L1 in serum. The presence of the three sulfonate groups on the outer perimeter of the pyrene moiety hampers the binding at the hydrophobic Sudlow sites as experimentally shown by relaxometric competition tests using classical binders for HSA hydrophobic sites (Figure S5). We surmised that potential binding sites for the hydrophilic pyrene substituent are represented by the positively charged groups of lysine or arginine residues present on the protein surface. To get more insight into this possibility, we undertook a relaxometric study of Gd-L1 with polylysine (MW between 30 and 70 kDa) (Figure 3). The observed behavior suggested that there is a binding interaction with the positively charged NH_3_^+^ groups, although the highly flexible random coil structure adopted at neutral pH by the macromolecule may limit the potential enhancement.^56^ Upon increasing the pH of the solution, the relaxivity increases as a consequence of the elongation of the molecular reorientational time, τ_R_, of the supramolecular adduct following the transition from the random coil to the α helix structure.^57^ In fact, the decrease in protonated ε-NH_3_^+^ moieties allows the polylysine to acquire a less flexible ε-helix-based structure which, in turn, results in an enhanced relaxivity. This effect is obviously counteracted by the decrease of protonated ε-NH_3_^+^ moieties, but likely the lengthening of the reorientational time is more important in determining the observed relaxation enhancement. At any pH, one cannot envisage the occurrence of pockets on the macromolecule structure where the pyrene substituent could fit; i.e., one has to convey that the occurring binding interaction involves only the cationic NH_3_^+^ groups and the trisulfonated pyrene residue of Gd-L1. The n*K*_a_ is high (1 × 10^5^ M^–1^) and one may surmise that in this polypeptide, any binding site is independent of the others. Obviously, the high number of binding sites (up to 342 available lysine residues in the used polymer) suggests that the affinity at any individual lysine residue is relatively small.

Next, it was deemed of interest to assess whether other serum proteins might contribute to the increased relaxivity of Gd-L1 in this medium as compared to the buffered HSA solution, in particular at lower concentrations of the paramagnetic complex. Based on the reasoning that electrostatic binding interactions may be responsible for the observed effect, we looked at the relaxivity enhancements of Gd-L1 in γ-globulin-containing media. This class of proteins is characterized by a basic isoelectric point and therefore appeared as a good candidate for the sought interactions. The titration of a buffered solution of γ-globulins with increasing amounts of Gd-L1 resulted in a profile that definitively paralleled the behavior observed in serum, thus suggesting that γ-globulins are likely the macromolecular system contributing to the relaxation enhancement detected in serum, in addition to the largest one provided by HSA.

To determine whether the molecular interactions that cause relaxation enhancement in serum are also active in the ECM, we carried out *r*_1_ measurements of Gd-L1 in an ECM-mimicking medium. For this purpose, we used Hystem (a mixture of polysaccharides, proteins, and other components), which is commercially available in a composition with or without collagen.^46^ Upon comparing the relaxivities in the two Hystem media, it is evident that Gd-L1 displays a higher relaxivity in the presence of collagen, whereas for the parent Gd-HPDO3A such a difference is much less pronounced (Figure 4). Collagen fibers have many lysine residues only partially used for the generation of the matrix network (through the conjugation with saccharides and other collagen molecules or other proteins). Thus, one may conclude that collagen in the ECM may represent a potential binding site for Gd-L1. Evidence for this hypothesis was gained by acquiring the *in vivo* MRI images of three organs (liver, spleen, kidneys) that are known to be highly fenestrated thus allowing an easy extravasation of Gd-L1 to the ECM. The signal enhancement (SI%) measured immediately after the i.v. administration of Gd-L1 in the three organs reached values of up to 6-fold higher than those observed for the parent Gd-HPDO3A (Figure 7). The choice to include Gd-HPDO3A as a control complex was made with the intention of comparing our system with a complex that would not interact with proteins of serum and other biological matrices. At first glance, the *in vivo* results were unexpected, since the MRI scans were acquired at 7 T (i.e., at a field strength that does not allow to take advantage of the remarkable relaxation enhancement whose maximum is at around 1 T). We think that the observed behavior reflects an increased uptake of Gd-L1 in the extravascular space that can be ascribed to the setup of numerous binding interactions with the proteins in the extravascular/extracellular space, despite the limited relaxivity enhancement observed *in vitro* under the high field at which the images were acquired. Thus, the *in vitro* results appear fully confirmed by the *in vivo* observations showing that the weak electrostatic interactions, likely exploiting the NH_3_^+^ moieties of lysine/arginine residues on different endogenous proteins, are a powerful source for the generation of an improved response in contrast-enhanced (CE)-MR images.

The enhanced *in vivo* response of Gd-L1 showed limited persistence (Figure 7). Indeed, the effect largely disappeared already after 10–15 min and the detected SI% for Gd-L1 became similar to the one shown by Gd-HPDO3A. The observed behavior can be accounted for in terms of the “washing out” of Gd-L1 from ECM. In these tissues the ECM space is limited and the ratio between the amount of extravasated Gd-L1 and the number of interaction sites on the endogenous macromolecules may favor the involvement of the strongest binding sites.

The excretion kinetics of Gd-L1 from the bloodstream was found to be similar to the one shown by Gd-HPDO3A, as ascertained by the Gd elimination curves from blood (Figure S9).

Therefore, the exploitation of the weak, although abundant, NH_3_^+^-involving interactions yielded good signal enhancement without affecting the excretion characteristics of Gd-L1. The limited persistence of the strong enhancement may be considered with interest as the CE-MR images are commonly acquired immediately after the administration of the contrast agent.

## Conclusions

In summary, this work shows that Gd-L1 is a good model for investigating new routes to electrostatic binding interactions with NH_3_^+^ exposing macromolecules to pursue marked relaxation enhancements and increased signals in *T*_1_-weigthed MR images. The large increase in signal enhancement detected at short times after the i.v. administration suggests that Gd-L1 can be used at doses that may be markedly lower than those applied with the currently available GBCAs. Therefore, Gd-L1 can be seen as the prototype of a novel class of GBCAs (or more in general of paramagnetic metal complexes), i.e., small-sized, stable, and hydrophilic paramagnetic complexes, able to interact with endogenous proteins in the vascular system and in the ECM. The herein-reported achievements offer new insights into the design of paramagnetic MRI contrast agents endowed with high relaxivity and a controlled biodistribution and excretion pathway.

## Experimental Section

### Synthesis

Chemicals were purchased from Sigma Aldrich Co. NMR spectra were recorded at 310 K on a Bruker AVANCE 600 spectrometer operating at 14 T (corresponding to 600 and 150 MHz ^1^H and ^13^C Larmor frequencies, respectively). Analytical and preparative HPLC–MS was carried out on a Waters Auto Purification system (3100 Mass Detector, 2545 Pump Gradient Module, 2767 Sample Manager, and 2998 PDA detector). The purity was double checked by analytical HPLC using an Atlantis C18, 3.5 μm, 4.6 mm × 150 mm column and 0.1% TFA in water (solvent A) and acetonitrile (solvent B); applying a gradient of CH_3_CN in H_2_O (0.1%TFA) from 5 to 80% in 15 min and from 80 to 100% in 5 min, flow 1 mL min^–1^ (method 1). pH measurements were made by using an AS pH meter equipped with a glass electrode.

### Synthesis of CM-pyranine

CM-pyranine (Trisodium 8-(methoxycarbonylmethoxy)pyrene-1,3,6-trisulfonate) was prepared following a reported procedure.^39^ A solution of HPTS (1.0 g, 1.96 mmol) in 20 mL of methanol was heated to reflux, followed by the addition of methyl bromoacetate (1.13 g, 0.68 mL, 7.2 mmol) and *N*,*N*-diisopropylethylamine (DIPEA) (0.70 g, 0.94 mL, 5.4 mmol) in 3 aliquots over 5 h. Reflux was maintained for three additional hours; the mixture was then filtered and the filtrate was evaporated, the residue was stirred in 20 mL of isopropanol for 30 min and collected by filtration. The recovered yellow powder was dissolved in water (40 mL) and purified on a Sephadex LH-20 column with water as the eluent. (0.6 g, 0.94 mmol, 48%). Methyl ester was quantitatively hydrolyzed with 2.4 M aqueous HCl at 90 °C. Evaporation of the reaction mixture followed by repeated lyophilization afforded pure CM-pyranine.

^1^H NMR (600 MHz, D_2_O): δ = 9.12 (s, 1H), 9.08 (d, 1H, *J* = 9.80), 9.00 (d, 1H, *J* = 9.60), 8.91 (d, 1H, *J* = 9.80), 8.89 (d, 1H, *J* = 9.60), 8.18 (s, 1H), 4.92 (s, 2H). ^13^C NMR (600 MHz, D_2_O): δ = 175.1, 154.0, 141.0, 137.0, 132.3, 131.7, 129.5, 127.6, 127.5, 126.9, 126.3, 125.4, 123.5, 123.0, 119.9, 68.0.

### Synthesis of AHPDO3A(*t*-Bu)_3_

AHPDO3A(*t*-Bu)_3_ was prepared following a reported procedure^38^ starting from 10-[3-[(benzyloxycarbonyl)amino]-2-hydroxypropyl]-1,4,7,10-tetra-azacyclododecan-1,4,7-triacetic acid tri-*t*-butyl ester, compound **1** (rif.1) by catalytic hydrogenolysis of *N*-benzyloxycarbonyl (Cbz). 10% Pd/C (225 mg) was added to a solution of compound **1** (1.5 g, 2.0 mmol) in MeOH (50 mL), and the reaction mixture was stirred under a hydrogen atmosphere for 6 h. The mixture was then filtered, and the solvent was evaporated to yield AHPDO3A(*t*-Bu)_3_ (0.35 g; 85%) as a pale-yellow oil.

^1^H NMR (600 MHz, CDCl_3_): δ = 3.96 (br, 1H), 3.46- 3.24 (br, 2H), 3.16 (br, 2H), 3.08–2.88 (br, 4H), 2.64–2.2 (enveloped signal, 12H), 2.18–2.00 (br, 6H), 1.40 (s, 27H). ^13^C NMR (600 MHz, CDCl_3_): δ = 175.0, 174.4, 174.0, 84.4, 69.5, 58.5, 57.8, 54.7, 52.7, 50.9, 48.2, 30.29. ESI-MS *m*/*z* calculated for C_29_H_57_N_5_O_7_: [M + H]^+^ 588.43, found 588.78.

### Synthesis of HPDO3A(*t*-Bu)_3_-trisulfonated Pyrene

HBTU (0.247 g, 0.65 mmol) and DIPEA (0.4 g, 3.2 mmol) were added to a CM-pyranine (0.380 g, 0.65 mmol) solution in dimethylformamide (10 mL). The resulting solution was stirred at room temperature for 20 min. Then, AHPDO3A(*t*-Bu)_3_ (0.380 g, 0.65 mmol) was added, and stirring was continued for 4 h. The addition of diethyl ether (50 mL) resulted in the precipitation of a yellow solid, which was isolated by centrifugation and washed with diethyl ether (10 mL) to give the crude product. The product was dissolved in water and purified on Amberchrome CG 161 resin with a H_2_O/CH_3_CN gradient as the eluent: the pure fractions were evaporated and freeze-dried. (0.46 g; 62%). The purity was verified by analytical HPLC using method 1 with UV detection at 220 and 400 nm (retention time = 10.48 min, purity >85%, λ = 220 nm).

^1^H NMR (600 MHz, D_2_O): δ = 9.11 (s, 1H), 9.05 (d, 1H, *J* = 9.75), 9.03 (d, 1H, *J* = 9.75), 8.92 (d, 1H, *J* = 9.83), 8.67 (br, 1H), 8.18 (s, 1H), 4.86 (br, 2H), 3.92 (br, 1H), 3.77–3.44 (br, 4H), 3.40–2.14 (enveloped signal, 22H), 1.16 (s, 27H). ^13^C NMR (600 MHz, D_2_O): δ = 175.9, 175.4, 174.9, 170.1, 165.1, 153.4, 145.8, 142.8, 142.6, 130.7, 130.3, 128.8, 127.7, 127.2, 126.7, 123.8, 123.4, 122.6, 111.8, 83.9, 70.8, 58.5, 58.0, 54.7, 52.6, 51.3, 47.1, 30.4. ESI-MS *m*/*z* calculated for C_47_H_67_N_5_O_18_S_3_: [M + H]^+^ 1087.25, found 1087.84.

### Synthesis of HPDO3A-trisulfonated Pyrene (L1)

To a solution of HPDO3A(*t*-Bu)_3_-trisulfonated pyrene (0.3 g, 0.26 mmol) in dichloromethane (20 mL), cooled in an ice bath, was slowly added trifluoroacetic acid (5.0 mL). The solution was stirred for 16 h at room temperature, and then diethyl ether (60 mL) was slowly added to give a yellow solid, which was filtered and washed with ether (5 × 30 mL). The solid was dissolved in water (30 mL) and purified on Amberchrome CG 161 resin with a H_2_O/CH_3_CN gradient as the eluent. The pure fractions were evaporated and freeze-dried. (0.11 g; 46%). The purity was verified by analytical HPLC using method 1 with UV detection at 220 and 400 nm (retention time = 4.69 min, purity = 94%, λ = 220 nm). ESI-MS *m*/*z* calculated for C_35_H_43_N_5_O_18_S_3_: [M + H]^+^ 918.18, found 918.67, [M + 2H]^2+^ 459.59, found 459.74 (Figure S1). ^1^H NMR (600 MHz, D_2_O): δ = 9.12 (s, 1H), 9.01 (d, 1H, *J* = 9.74), 8.96 (d, 1H, *J* = 9.60), 8.89 (d, 1H, *J* = 9.66), 8.63 (d, 1H, *J* = 9.60), 8.09 (s, 1H), 4.95 (s, 2H), 3.90 (br, 1H), 3.79 (br, 1H), 3.32–2.12 (enveloped signal, 25H). ^13^C NMR (600 MHz, D_2_O): δ = 173.6, 154.2, 141.8, 138.1, 132.3, 131.7, 129.7, 127.9, 127.7, 127.2, 126.3, 126.2, 124.07, 123.5, 112.5, 70.7, 58.3, 57.0, 56.7, 55.0, 53.5, 50.7, 44.8 (Figure S2).

### Synthesis of Ln-L1 (Ln = Gd, Eu)

The L1 ligand (0.1 g, 0.11 mmol) was dissolved in water (10 mL) and the pH was adjusted to 6.5 by adding 1 M NaOH. Then, 0.4 mL of a 0.25 M solution of LnCl_3_ in water was slowly added while the pH value was maintained at 6.7 with NaOH. The mixture was stirred at room temperature for 16 h. The product was purified on Amberchrome CG 161 resin with a H_2_O/CH_3_CN gradient as the eluent. The pure fractions were evaporated and freeze-dried. (0.088 g; 82% for Gd-L1 and 0.090 g; 77% for Eu-L1). The purity was verified by analytical HPLC using method 1 with UV detection at 220 and 400 nm.

Gd-L1: retention time = 3.36 min, purity = 95%, λ = 220 nm; ESI-MS *m*/*z* calculated for C_35_H_40_GdN_5_O_18_S_3_: [M + H]^+^ 1073.08, found 1073.41; [M + 2H]^2+^ 537.04, found 537.31; [M – H]^−^ 1071.08, found 1072.40; [M – 2H]^2–^ 535.04, found 535.42 (Figure S3).

Eu-L1: retention time = 3.46 min, purity = 95%, λ = 220 nm; ESI-MS *m*/*z* calculated for C_35_H_40_EuN_5_O_18_S_3_: [M + H]^+^ 1068.07, found 1068.06; [M – H] 1066.07, found 1066.06; [M – 2H]^2–^ 532.03, found 532.59.

### Octanol–Water Partition Coefficient (log *P*)

3 mL of an aqueous solution of Gd-L1 (0.5 mM) was added to 3 mL of octanol. The resulting mixture was vigorously shaken in a centrifuge tube for 30 min and then transferred to a separation funnel. The solution was allowed to equilibrate for 24 h. After separating, the aqueous fraction was analyzed using a Waters Alliance 2695 HPLC system with Waters 2998 Photodiode Array (PDA) detector equipped with an Atlantis C18 Column, 3.5 μm, 4.6 mm × 150 mm and 7 mM CH_3_COONH_4_, pH = 7 (solvent A) and acetonitrile (solvent B) as the eluent. Elution was carried out with 100% of solvent A for 2 min and then 0% to 70% gradient of solvent B into A over 15 min at a 1 mL/min flow rate. The detection wavelength was set at 254 nm. The sample solution was injected into a volume of 20 μL. The HPLC was calibrated with Gd-L1 solutions of 0.12–1 mM (correlation coefficient of *R*^2^ = 1), *t*_R_^Gd–L1^ = 6.7 min. The quantity of Gd-L1 present in the organic layer was calculated as a difference from the total quantity originally introduced. The log *P* value was calculated by the following equation log *P* = log(*C*_octanol_/*C*_water_) where *C*_octanol_ and *C*_water_ refer to the concentrations of Gd-L1 in *n*-octanol and in water, respectively.

### Relaxometric Measurements

The observed longitudinal relaxation rate (*R*_1_ = 1/*T*_1_) values were determined by inversion recovery at 21.5 MHz and 25 °C using a Stelar SpinMaster spectrometer (Stelar s.r.l, Mede (PV), Italy). Temperature was controlled with a Stelar VTC-91 airflow heater and the temperature inside the probe was checked using a calibrated RS PRO RS55-11 digital thermometer. Data were acquired using a recovery time ≥5 × *T*_1_ and with 2 scans per data point. The absolute error in the *R*_1obs_ measurements was less than 1%.

The concentration of the solutions used for the relaxometric characterization was determined by using the previously reported relaxometric method.^57^

The interaction of Gd-L1 with HSA, γ-globulins, and polylysine was studied using the well-established proton relaxation enhancement (PRE) method.^42^ Namely, the apparent binding constant (*K*_a_) and the relaxivity of the resulting adduct (*r*_b_) were determined by measuring *R*_1_ values of Gd-L1 solutions at a fixed Gd concentration, as a function of increasing concentration of macromolecule, in PBS at 298 K, 21.5 MHz, and pH 7.4.

The relaxometric competition tests for the hydrophobic sites of HSA (Figure S5) were carried out by measuring *R*_1_ values of solutions containing Gd-L1 (0.1 mM), HSA (0.6 mM), and several HSA-binders (0.6 mM), in PBS at 298 K, 21.5 MHz, and pH 7.4. Warfarin and iodipamide were used for the Sudlow site I (subdomain IIA), ibuprofen for the Sudlow site II (subdomain IIIA), and methyl orange for subdomain IB.

### ^1^H-NMRD Profiles

NMRD profiles were obtained by using a Stelar SmartTracer FFC NMR relaxometer from 0.01 to 10 MHz. Additional data in the 20–80 MHz frequency range were obtained with a High Field Relaxometer (Stelar) equipped with an HTS-110 3T Metrology cryogen-free superconducting magnet and a Bruker WP80 NMR electromagnet (21.5–80 MHz), both equipped with a Stelar VTC-91 for temperature control; the temperature inside the probe was checked with a calibrated RS PRO RS55-11 digital thermometer. Aqueous and human serum solutions of the complex were measured at 298 K. The NMRD profile data were fitted using the Solomon–Bloembergen–Morgan and Freed’s models.

### O^17^-*R*_2_ vs T NMR Measurements

O^17^-*R*_2_ vs T NMR measurements were performed at 14.1 T on a Bruker Avance 600 spectrometer at variable temperatures, with a D_2_O sealed capillary for sample locking inside the tube. The 20 mM Gd-complex solutions were enriched with 1% H_2_^17^O (Cambridge Isotope). The width at half-maximum (Δω_dia_) of the H_2_^17^O signal in pure water was measured over the investigated temperature range and subtracted from the width at half-maximum (Δω_Gd_) of the tested Gd-complexes solutions. Then, *R*_2_ was calculated as follows: *R*_2_ = π[Δω_Gd_ – Δω_dia_].

### High-Resolution ^1^H NMR

The Eu-L1 complex was dissolved in D_2_O (12 mM) and the pH was adjusted by the addition of DCl or KOD and tested with a glass electrode connected to an AsInstruments pH meter. The ^1^H NMR spectra were recorded at 14.1 T on a Bruker Avance 600 spectrometer. The temperature was controlled with a Bruker thermostat units.

The HPTS binding affinity toward HSA was investigated by acquiring ^1^H NMR spectra of HPTS 0.6 mM PBS solutions in D_2_O, upon the addition of increasing concentrations of HSA (0–2.1 mM). 3-(Trimethylsilyl)-1-propanesulfonic acid-*d*_6_ sodium salt (DSS) was used as the NMR reference standard. The chemical shift variation of aromatic proton 2 of HPTS was plotted as a function of HSA concentration (Figure S6).

### Animal Handling

For the *in vivo* imaging experiments, 8–10-week-old male Balb/c mice (Charles River Laboratories, Calco, Bergamo, Italy) were used. The mice were bred at the animal house of the Molecular Imaging Center (MBC) at the University of Turin. They were kept in standard housing conditions with standard rodent chow, water available *ad libitum*, and a 12-h light/dark cycle.

All procedures involving animals were performed in accordance with national and international laws on the use of experimental animals (L.D. 26/2014; Directives 2010/63/EU) under Ministerial Authorization (project Research Number 888/2021-PR protocol CC652.167.EXT.53).

### MRI Acquisition and Data Analysis

For MRI experiments, mice were anesthetized by intramuscular injection of a mixture of 20 mg/kg tiletamine/zolazepam (Zoletil 100, Virbac, Milan, Italy) and 20 mg/kg + 5 mg/kg xylazine (Rompun; Bayer, Milan, Italy). Permanent vein access was obtained by inserting a PE10 catheter into the tail vein.

A glass tube containing a standard solution was used as an internal reference. It was located in the field of view in the proximity of the mouse body.

MR images were acquired, pre- and post-injection of Gd-complexes, at 7.1 T by using a Bruker Avance 300 spectrometer equipped with a Micro 2.5 microimaging probe, at room temperature (ca. 21 °C).

Mice were intravenously injected with either 0.15 mmol/kg Gd-L1 or 0.15 mmol/kg ProHance.

*T*_2w_ images were acquired by using a standard *T*_2w_ RARE (Rapid Acquisition with Refocused Echoes) sequence with the following parameters (TR = 5000 ms, TE = 5.5 ms, RARE factor = 32, FOV = 3.5 × 3.5 cm^2^, slice thickness = 1 mm, matrix 128 × 128).

*T*_1w_ images were acquired immediately after the injection of Gd-L1 or ProHance by using a standard *T*_2w_-MSME (multislice multiecho) sequence with the following parameters (TR = 200 ms, TE = 3.3 ms, number of averages = 6, FOV = 3.5 × 3.5 cm^2^, slice thickness = 1 mm, matrix 128 × 128, resolution 0.273 × 0.273 mm/pixel).

ROIs were manually drawn inside the organs of interest (spleen, liver, kidneys) and the mean of signal intensity (at least 5 ROIs in different slices) were calculated.

Signal enhancement was calculated using the following formula:
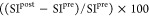
where SI^pre^ and SI^post^ are the signal intensities in *T*_1w_ images before and after the injection of Gd-complexes, respectively, upon normalization for the signal in the reference tube.

### Assessment of Blood Elimination by ICP-MS

The pharmacokinetics of intravenously administered Gd-L1 and ProHance were assessed by ICP-MS quantification of the Gd content in plasma. For this purpose, after the intravenous injection of 0.15 mmol/kg of Gd-L1 or ProHance to healthy mice (*n* = 3), blood was collected from mice tail veins at variable time points (*t* = 5 min, 10 min, 15 min, 30 min, 1 h, and 4 h). Before ICP-MS analysis, blood samples were digested with concentrated HNO_3_ (70%) under microwave heating (Milestone MicroSYNTH Microwave laboratory station, Balgach, Switzerland, equipped with an optical fiber temperature control and HPR-1000/6 M high-pressure reactor, Milestone, Bergamo, Italy). After the digestion, 3 mL of ultrapure water was added to each sample. The specimens were then subjected to ICP-MS analysis (Element-2; Thermo-Finnigan, Rodano (MI), Italy) to measure the concentration of Gd with respect to standard curves. Results were reported as the Gd micromolar concentration as a function of collection time.

## Supporting Information Available

HPLC-MS and NMR data of the synthesized compounds, PRE titrations, relaxometric competitive tests, transmetalation competition assay, high-resolution NMR spectra of Eu-L1, ^1^H NMR measurement of *K*_a_ to HSA for HPTS, and blood elimination curves from blood containing Gd-L1 and ProHance (PDF)

## Abbreviations

CA: contrast agent
CE: contrast enhanced
DIPEA: *N,N*-diisopropylethylamine
DMF: *N*,*N*-dimethylformamide
PBS: phosphate buffer solution
GBCA: gadolinium-based contrast agent
MRI: magnetic resonance imaging
HPLC: high performance liquid chromatography
HBTU: *O*-(benzotriazol-1-yl)-*N*,*N*,*N*′,*N*′-tetramethyluroniumhexafluorophosphate
HPTS: 8-hydroxypyrene-1,3,6-trisulfonate
ICP: inductively coupled plasma
MS: mass spectrometry
MW: molecular weight
PRE: proton relaxation enhancement
*r*_1_: longitudinal relaxivity
*R*_1_: longitudinal relaxation rate
*R*_2_: transverse relaxation rate
SI: signal enhancement
ROI: region of interest
TFA: trifluoroacetic acid
