# Supplementary material for: Seeking for Innovation with Magnetic Resonance Imaging Paramagnetic Contrast Agents: Relaxation Enhancement via Weak and Dynamic Electrostatic Interactions with Positively Charged Groups on Endogenous Macromolecules
Source: J Am Chem Soc. 2023 Dec 28;146(1):134–44. doi: 10.1021/jacs.3c06275 (PMC10785807; doi:10.1021/jacs.3c06275)
Supplement: Supplementary file 1 — ja3c06275_si_001.pdf [file ja3c06275_si_001.pdf]

Supporting Information

Seeking for Innovation with Magnetic Resonance Imaging Paramagnetic Contrast Agents: Relaxation Enhancement via Weak and Dynamic Electrostatic Interactions with Positively Charged Groups on Endogenous Macromolecules

Rachele Stefania,[§ a,b] Lorenzo Palagi,[§ a] Enza Di Gregorio,[a] Giuseppe Ferrauto,[a] Valentina Dinatale,[a] Silvio Aime,[c] and Eliana Gianolio [a *]

[§] R.S. and L.P. contributed equally to this work

[a] Department of Molecular Biotechnology and Health Sciences, University of Torino, Torino 10126, Italy

[b] Department of Science and Technological Innovation, University of Eastern Piedmont, Alessandria 15120, Italy

[c] IRCCS SDN SYNLAB, Napoli 80142, Italy

Correspoding author:

Eliana Gianolio

Department of Molecular Biotechnology and Health Sciences, University of Torino, P.zza Nizza 44, 10126 Torino, Italy

Tel. +390116706475

e-mail: Eliana.gianolio@unito.it

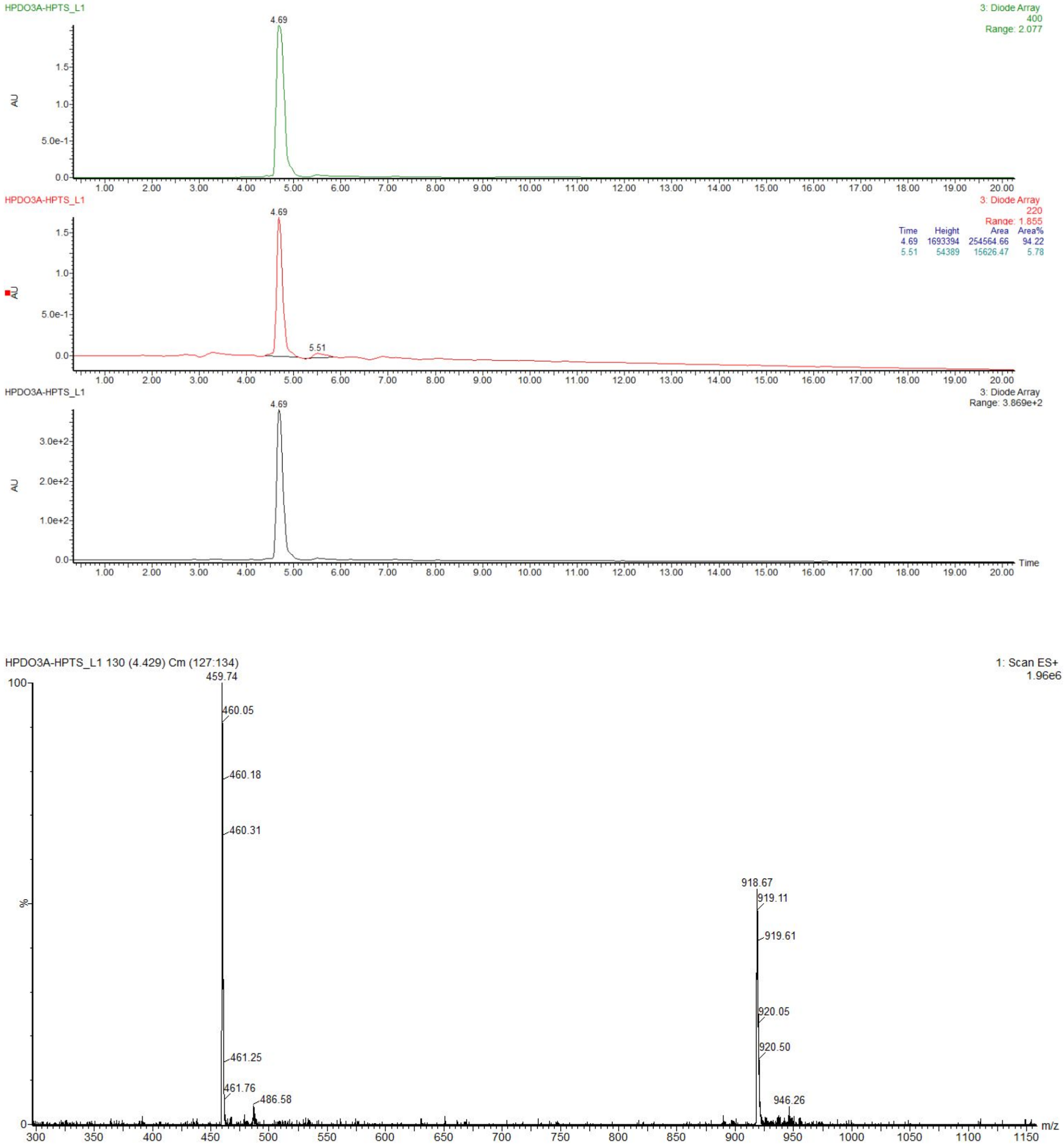

Figure S1. Top: HPLC chromatogram of L1 (diode array 200-400 nm, λ=220 nm, λ=400 nm). Bottom: mass spectrum ESI(+).

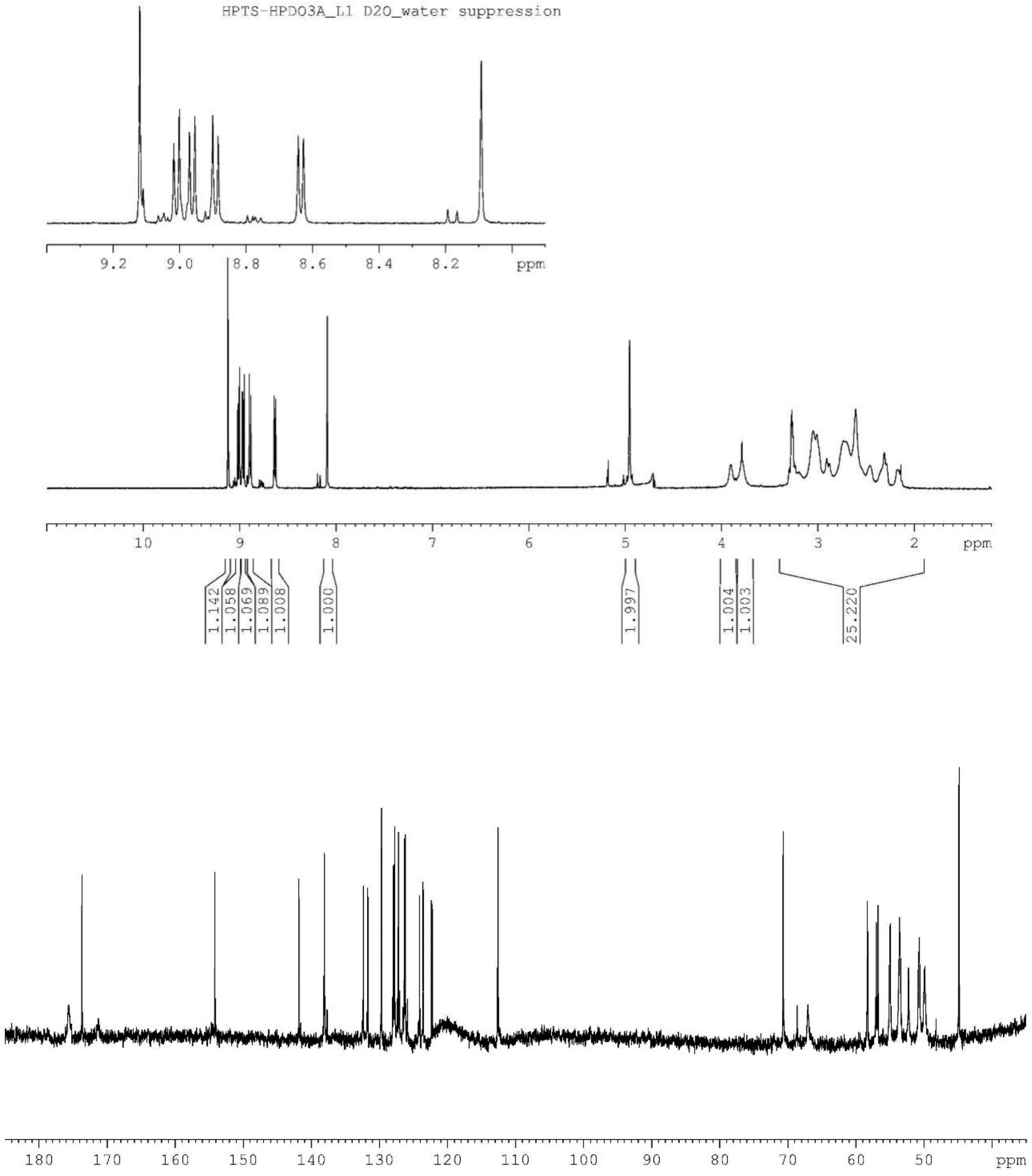

Figure S2. ^{1}H-NMR (600 MHz, D_2O, water suppression, 37 °C) and 13C-NMR (600 MHz, D_2O, 37 °C) of L1.

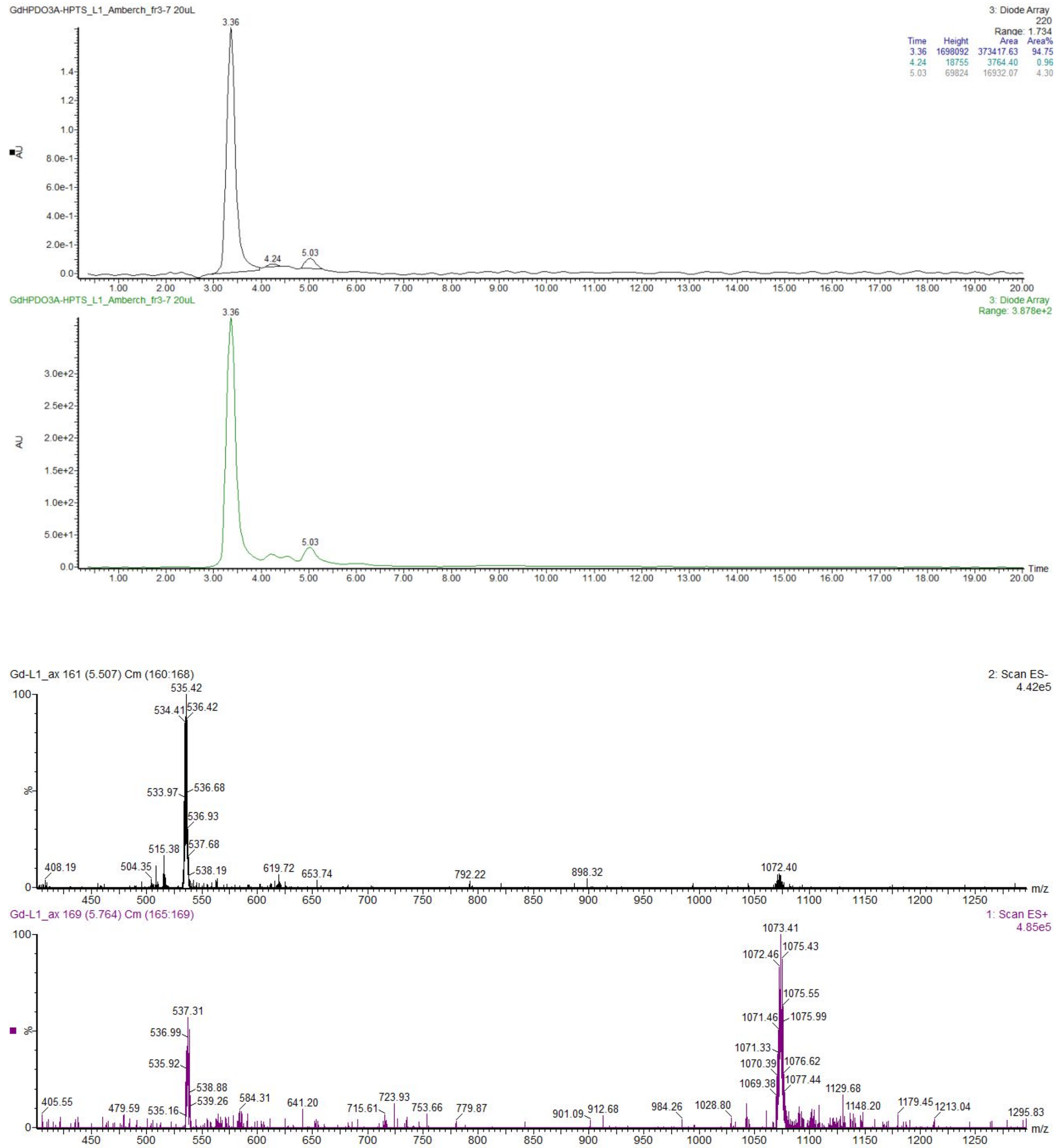

Figure S3. Top: HPLC chromatogram of Gd-L1 (diode array 200-400 nm, λ=220 nm,). Bottom: mass spectrum ESI(-) and ESI(+).

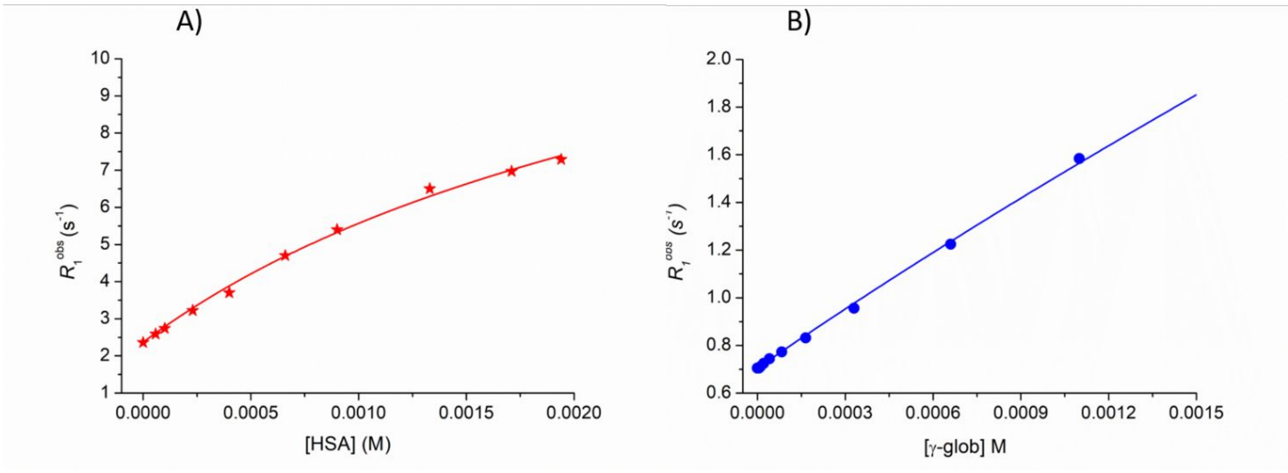

Figure S4. Proton Relaxation Enhancement (PRE) titrations of solutions of **A)** Gd-L1 (0.28 mM) with increasing amounts of Human Serum Albumin (HSA) and **B)** Gd-L1 (0.05 mM) with increasing amounts of γ-globulins (298 K, 21.5 MHz, in PBS).

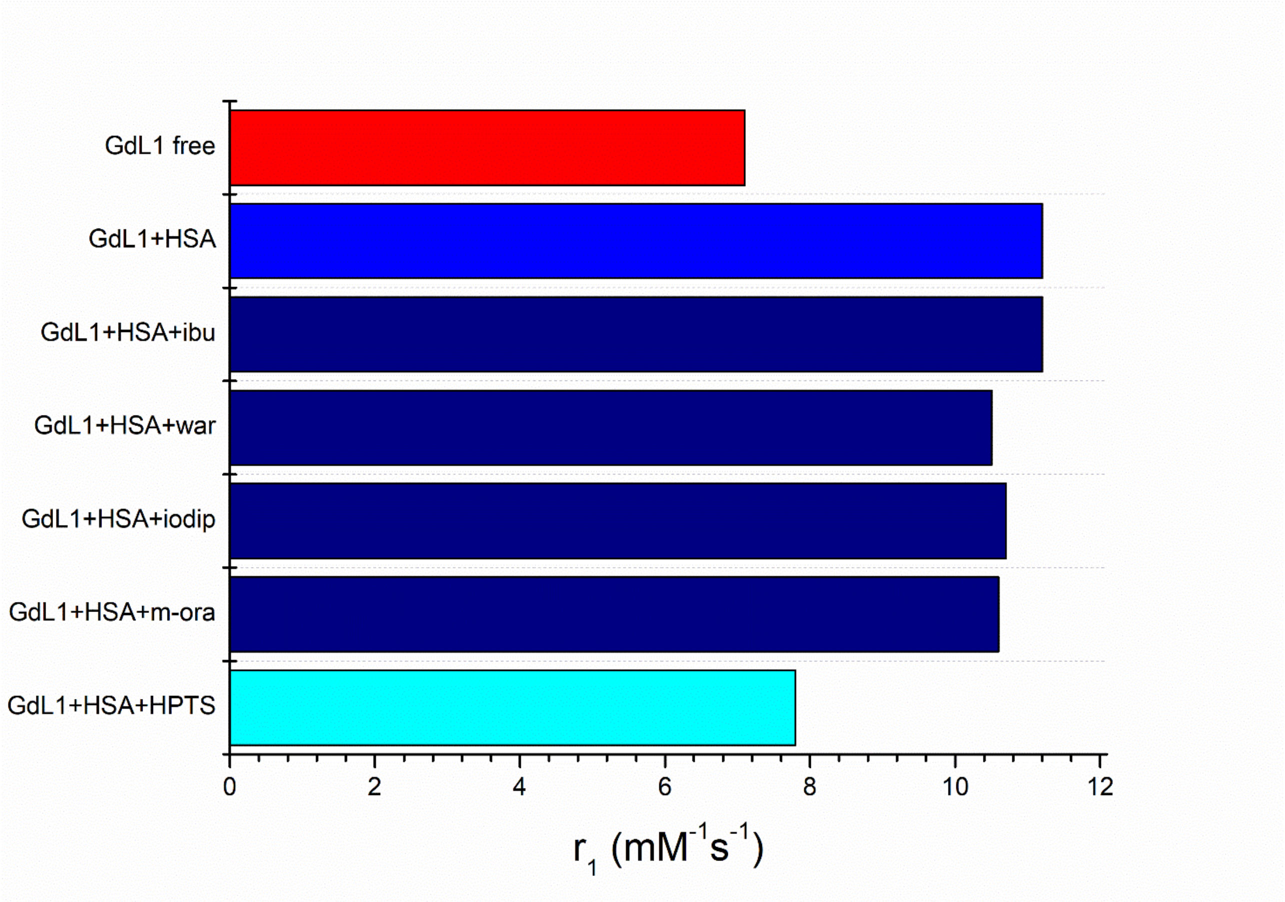

Figure S5. Proton millimolar relaxivities (r_1) of Gd-L1 (0.1 mM) in its free form, in the presence of human serum albumin (HSA, 0.6 mM) and in the presence of HSA (0.6 mM) and binders (0.6 mM) for the principal hydrophobic sites on albumin (ibuprofen (subdomain IIIA), iodipamide and warfarin (subdomain IIA) and methyl-orange (subdomain IB)) or HPTS (298 K, 21.5 MHz, in PBS).

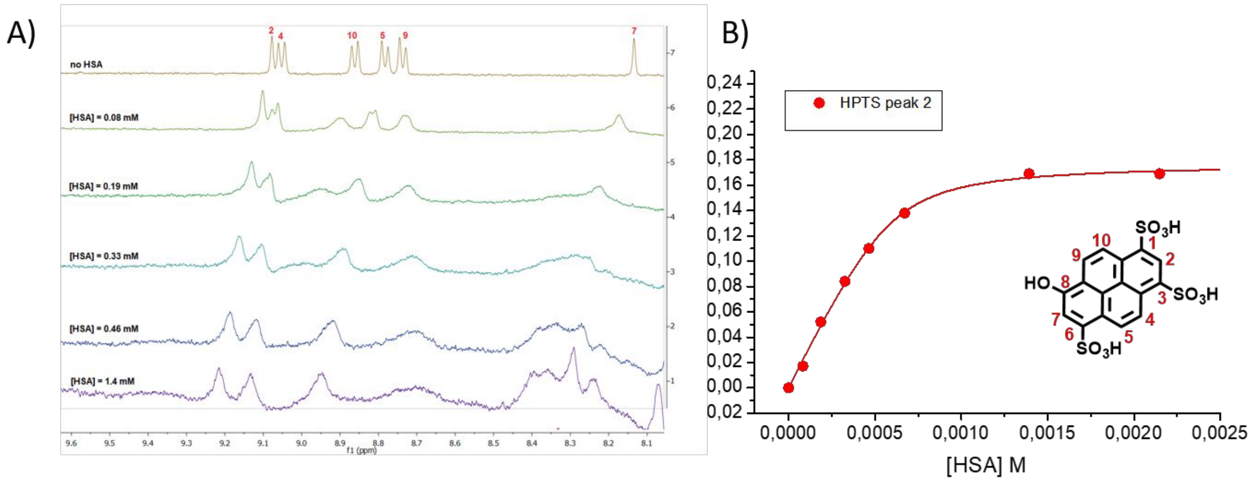

Figure S6: ^{1}H-NMR spectra of HPTS 0.6 mM upon addition of increasing concentrations of HSA (0-2.1 mM) (A) and chemical shift variation of peak 2 of HPTS as a function of HSA concentration (B) (600 MHz, PBS in D_2O, water suppression, 298K).

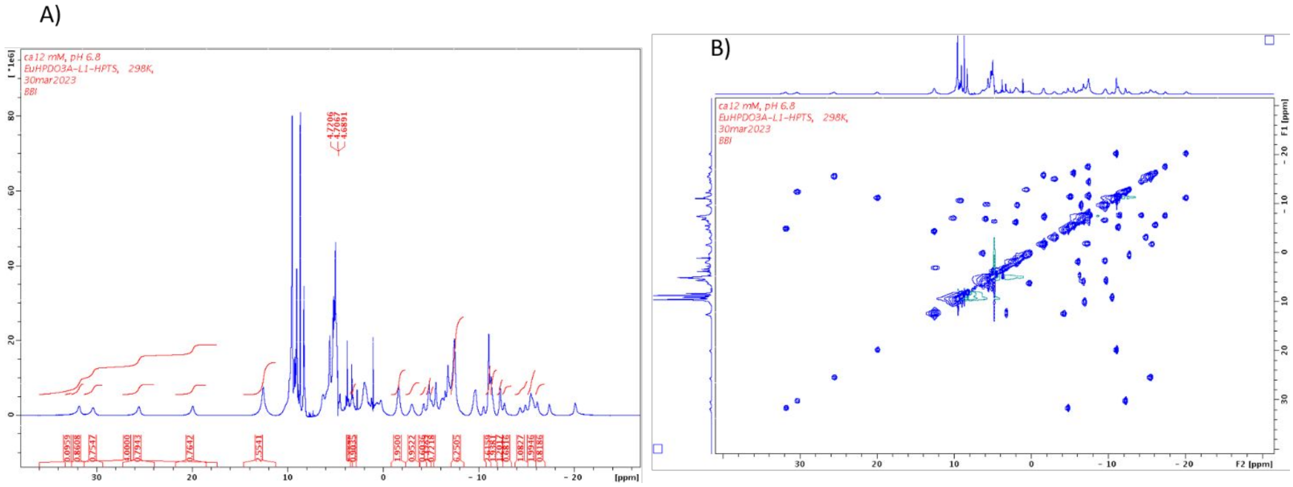

Figure S7: ^{1}H-NMR 1-D (A) and 2-D EXSY (B) spectra of 10 mM Eu-L1 (600 MHz, D_2O, water suppression, 298K, pH 6.8).

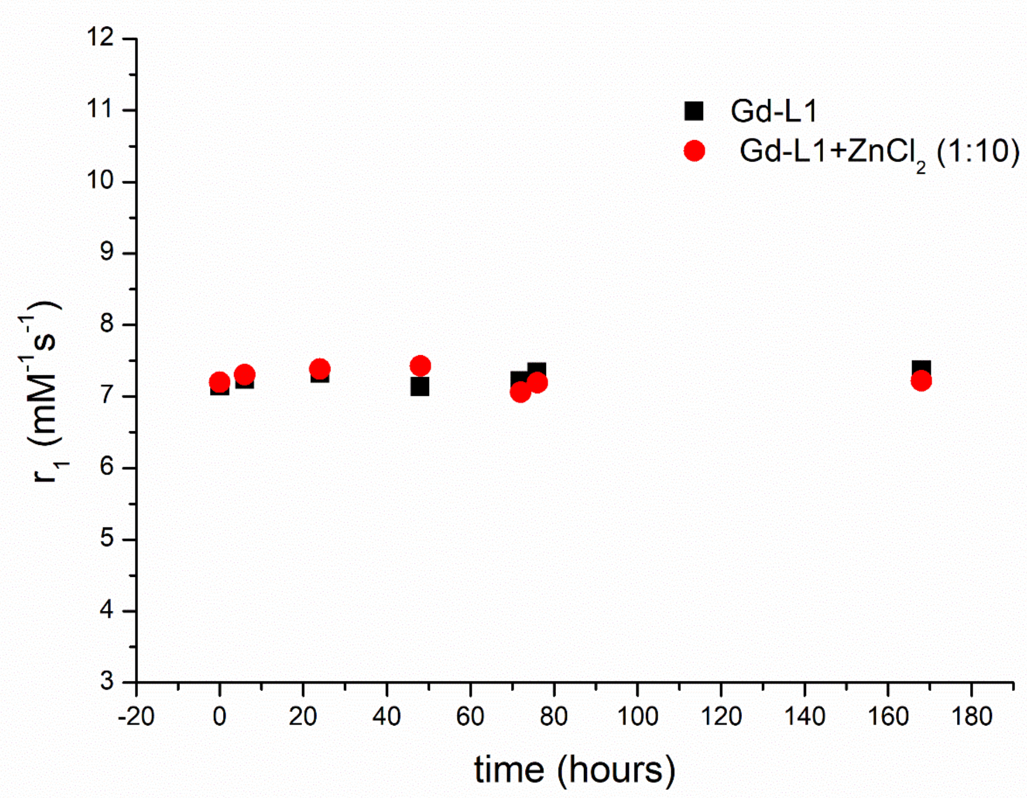

Figure S8: Transmetallation of GdL1 incubated with 10 eq. of zinc in 50 mM phosphate buffer at 310 K for 7 days, followed through the measure of relaxivity at 298 K, pH 7.4, at 0.47 T.

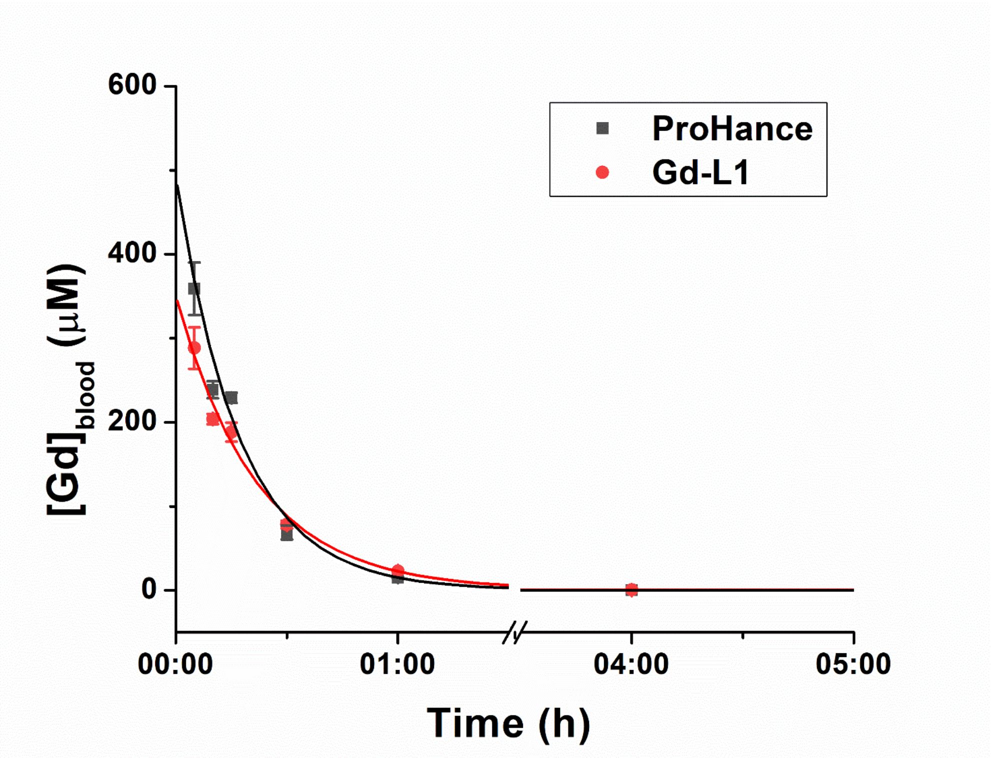

Figure S9: Blood elimination curves of Gd-L1 and ProHance upon the intravenous administration of 0.15 mmol/kg of contrast agent in healthy mice. The area under the curve (AUC) was computed for the elimination curves of GdL1 and Prohance. The calculated values were as follows: AUC = 5.832 for Prohance and AUC = 5.315 for Gd-L1. We don't consider worth of note this difference in the context of the, unexpected, fast clearance of Gd-L1 even if slight variations in the acquisition of the two experimental datasets could contribute to the observed difference.
